# Supplementary material for: When I am sixty-four… evaluating language markers of well-being in healthy aging narratives
Source: PLoS One. 2024 Apr 24;19(4):e0302103. doi: 10.1371/journal.pone.0302103 (PMC11042717; doi:10.1371/journal.pone.0302103)
Supplement: S1 File — (DOCX) [file pone.0302103.s001.docx]

**Supporting Information**

**Section A: Materials & Measures**

Writing instructions (translated to English):

For the next 15 minutes, we ask you to write down your deepest thoughts and feelings about

**“aging well”.** What does **“aging well”** mean to you personally?

Growing older is a process that is relevant to all of us throughout our entire lifespan.

For many, aging well means being able to be and to do what really matters to them.

This includes having the conditions under which they can pursue personally relevant goals and activities. What is **most important** to you in the process of aging well? What aspects are important to you in the **social domain** or in terms of your **emotions and well-being**?

What role does **physical health** play? Please write freely, wherever the topic may lead you.

In your writing, you may also link your thoughts with previous **experiences** you have made with your family, with people close to you, or in your professional life. How have these experiences shaped you in terms of how you would like to be and how you currently are?

We ask you to please write **without interruption** for the entire **15 minutes** and do not

do not worry about spelling, grammar, punctuation, or writing style in any way.

Just write away.

*(adapted from Expressive Writing Paradigm, see e.g., Pennebaker, 1997).*

Original instructions (in German):

In den nächsten 15 Minuten bitten wir Sie Ihre tiefsten Gedanken und Gefühle über

**„gutes Altern“** aufzuschreiben. Was bedeutet **„gut zu Altern“** für Sie persönlich?

Älter werden ist ein Prozess, der für uns alle über die gesamte Lebensspanne von

Bedeutung ist. Für viele bedeutet positives Altern das sein und tun zu können, was

einem wirklich wichtig ist. Dazu gehört Bedingungen zu haben, unter welchen man

persönlich relevante Ziele und Aktivitäten verwirklichen kann. Was ist Ihnen am

**Wichtigsten** für ein gutes Älterwerden? Welche Aspekte sind Ihnen im **sozialen**

**Bereich** oder hinsichtlich **Ihrer Stimmungen und Ihres Wohlbefindens** wichtig?

Welche Rolle spielt **körperliche Gesundheit**? Schreiben Sie frei aus sich heraus,

wo immer das Thema Sie auch hinführen mag.

Zudem können Sie das, was Sie schreiben auch in Bezug bringen mit **Erfahrungen**

mit Ihrer Familie, Ihnen nahestehenden Menschen oder Ihrem Beruf. Wie haben

diese Erfahrungen Sie geprägt in Bezug darauf, wie Sie gerne sein würden und wie

Sie aktuell sind?

Lassen Sie Ihren tiefen Gedanken und Gefühlen beim Schreiben **freien Lauf.**

Schreiben Sie bitte **ohne Unterbrechung** die gesamten **15 Minuten** durch und

kümmern Sie sich in keiner Weise um Rechtschreibung, Grammatik, Interpunktion

oder Schreibstil. Schreiben Sie einfach drauf los.

| **Table S1**  *Pre-Registered Questionnaire Scales* | | |
| --- | --- | --- |
| **Affective Well-being** | | |
| **Construct** | **Questionnaire name (abbreviation)** | **Reference** |
| Positive & Negative Affect | PANAS subscales:   - *Positive Affect (PA)* - *Negative Affect (NA)* | (Krohne et al., 1996; Watson et al., 1988) |
| Depressive Symptoms | PHQ-9 | (Kroenke et al., 2001) |
| Subjective (Mental) Health | SF-12 subscales:   - *mental health ^a^* - *emotional role functioning* | (Bullinger et al., 1995; Ware et al., 1996) |
| Subjective Quality of Life | WHO QoL BREF26 subscale:   - *mental QoL* - *WHOQoL16 (satisfaction with sleep item bref16)* | (Angermeyer et al., 2002; The Whoqol Group, 1998) |
| Subjective  Quality of Life in Older Age ^b^ | WHO QOL OLD (6-item short form): subscale:   - *worries about death* | (Conrad et al., 2014; Fang et al., 2012) |
| Sleep Quality | Jenkin’s sleep scale (JENKINS) | (Jenkins et al., 1988) |
| **Evaluative Well-Being** | | |
| Psychological Well-Being | PWS Ryff Subscales:   - *autonomy (autoRYFF)* - *environmental mastery (EMRYFF)* - *personal growth (PGRYFF)* - *purpose in life (PILRYFF)* - *self-acceptance (SARYFF)* | (Risch et al., 2013; Ryff, 1989) |
| Subjective (Physical) Health | SF-12 subscale:   - *Physical health* | (Bullinger et al., 1995; Ware et al., 1996) |
| Memory Complaints | Memory complaints | (Aschwanden et al., 2018)(Martin & Zimprich, 2003; Oswald & Fleischmann, 1995) |
| Subjective  Quality of Life | WHO QoL BREF26 subscales:   - *physical QoL^c^ (PHYS)* - *environment (ENVIR)* - *global QoL (OVERALL)* | (Angermeyer et al., 2002; The Whoqol Group, 1998) |
| Subjective  Quality of Life in Older Age ^b^ | WHO QOL OLD (6-item short form): subscales:   - *sensory functioning* - *autonomy* - *activities past/present/future* | (Conrad et al., 2014; Fang et al., 2012) |
| Functional  Quality of Life | fQoL-scale (fQoL) | (Eicher, 2014) |
| Individual Quality of Life | SEIQoL-DW (SEIQoL) | (Hickey et al., 1996) |
| Life Satisfaction (Current, Retrospective, Prospective) | “Skala zur Lebensbewertung” subscales:   - *current life satisfaction (LSSA)* - *retrospective life satisfaction (LSSP)* - *prospective life satisfaction (LSSZ)* | (Ferring et al., 1996) |
| Life Satisfaction | Satisfaction With Life Scale (SWLS; 5-item short form) | (Diener et al., 1985; Glaesmer et al., 2011) |
| **Social Well-being** | | |
| Psychological  Well-Being | PWS Ryff subscale:   - *positive relations with others (PRORYFF)* | (Risch et al., 2013; Ryff, 1989) |
| Subjective Health | SF-12 subscale:   - social functioning | (Bullinger et al., 1995; Ware et al., 1996) |
| Subjective  Quality of Life | WHO QoL BREF26 subscales:   - *social relationships (SOCIAL)* | (Angermeyer et al., 2002; The Whoqol Group, 1998) |
| Subjective  Quality of Life in Older Age ^b^ | WHO QOL OLD (6-item short form): subscale:   - *intimacy* - *social participation* | (Conrad et al., 2014; Fang et al., 2012) |
| Relationship  Satisfaction ^d^ | Dyadic Adjustment Scale (DAS-4) | (Dinkel & Balck, 2006; Sabourin et al., 2005) |
| Social Support | Berlin social support subcales:   - *perceived available instrumental support (BSSSI)* - *perceived available emotional support (BSSSE)* | (Schulz & Schwarzer, 2003) |
| Loneliness | UCLA loneliness scale (10-item version; UCLA) | (Döring & Bortz, 1993; Russell et al., 1980) |
| *Note.* Pre-registered questionnaire scales that were expected to load onto different well-being components in the language measurement model (see: <https://osf.io/jwkh9>).  ^a^ One item about “social functioning” was expected to load onto the “social well-being” factor  ^b^ Only answered by participants who were 60 years or older.  ^c^ One item about sleep satisfaction was expected to load onto “affective well-being”.  ^d^ Only answered by participants who were in a romantic relationship at the time of the study. | | |

**References**

Angermeyer, C., Kilian, R., & Matschinger, H. (2002). Deutschsprachige Version der WHO Instrumente zur Erfassung von Lebensqualität WHOQOL-100 und WHOQOL-BREFM. *Zeitschrift Für Medizinische Psychologie*, *11*(1), 44–48.

Aschwanden, D., Kliegel, M., & Allemand, M. (2018). Cognitive complaints mediate the effect of cognition on emotional stability across 12 years in old age. *Psychology and Aging*, *33*(3), 425–438. https://doi.org/10.1037/pag0000246

Bullinger, M., Kirchberger, I., & Ware, J. (1995). Der deutsche SF-36 Health Survey Übersetzung und psychometrische Testung eines krankheitsübergreifenden Instruments zur Erfassung der gesundheitsbezogenen Lebensqualität [The German SF-36 health survey translation and psychometric testing of a generic instrument for the assessment of health-related quality of life]. *Journal of Public Health*, *3*(1), 21–36. https://doi.org/10.1007/BF02959944

Conrad, I., Matschinger, H., Riedel-Heller, S., von Gottberg, C., & Kilian, R. (2014). The psychometric properties of the German version of the WHOQOL-OLD in the German population aged 60 and older. *Health and Quality of Life Outcomes*, *12*(1), 105. https://doi.org/10.1186/s12955-014-0105-4

Diener, E., Emmons, R. A., Larsen, R. J., & Griffin, S. (1985). The Satisfaction With Life Scale. *Journal of Personality Assessment*, *49*(1), 71–75. https://doi.org/10.1207/s15327752jpa4901_13

Dinkel, A., & Balck, F. (2006). Psychometrische Analyse der deutschen Dyadic Adjustment Scale. *Zeitschrift für Psychologie / Journal of Psychology*, *214*(1), 1–9. https://doi.org/10.1026/0044-3409.214.1.1

Döring, N., & Bortz, J. (1993). Psychometrische Einsamkeitsforschung: Deutsche Neukonstruktion der UCLA Loneliness Scale. [Psychometric research on loneliness: A new German version of the University of California at Los Angeles (UCLA) Loneliness Scale.]. *Diagnostica*, *39*(3), 224–239.

Eicher, S. (2014). *Quality of life in healthy old age: How it can be defined, measured and stabilized from a within-person perspective*. https://doi.org/10.5167/UZH-126799

Fang, J., Power, M., Lin, Y., Zhang, J., Hao, Y., & Chatterji, S. (2012). Development of Short Versions for the WHOQOL-OLD Module. *The Gerontologist*, *52*(1), 66–78. https://doi.org/10.1093/geront/gnr085

Ferring, D., Filipp, S.-H., & Schmidt, K. (1996). Die “Skala zur Lebensbewertung”: Empirische Skalenkonstruktion und erste Befunde zu Reliabilität, Stabilität und Validität. [The Life Satisfaction Scale: Construction and findings on reliability, stability, and validity.]. *Zeitschrift Für Differentielle Und Diagnostische Psychologie*, *17*(3), 141–153.

Glaesmer, H., Grande, G., Braehler, E., & Roth, M. (2011). The German Version of the Satisfaction With Life Scale (SWLS): Psychometric Properties, Validity, and Population-Based Norms. *European Journal of Psychological Assessment*, *27*(2), 127–132. https://doi.org/10.1027/1015-5759/a000058

Hickey, A. M., Bury, G., O’Boyle, C. A., Bradley, F., O’Kelly, F. D., & Shannon, W. (1996). A new short form individual quality of life measure (SEIQoL-DW): Application in a cohort of individuals with HIV/AIDS. *BMJ*, *313*(7048), 29–33. https://doi.org/10.1136/bmj.313.7048.29

Jenkins, C. D., Stanton, B.-A., Niemcryk, S. J., & Rose, R. M. (1988). A scale for the estimation of sleep problems in clinical research. *Journal of Clinical Epidemiology*, *41*(4), 313–321. https://doi.org/10.1016/0895-4356(88)90138-2

Kroenke, K., Spitzer, R. L., & Williams, J. B. W. (2001). The PHQ-9: Validity of a brief depression severity measure. *Journal of General Internal Medicine*, *16*(9), 606–613. https://doi.org/10.1046/j.1525-1497.2001.016009606.x

Krohne, H. W., Egloff, B., Kohlmann, C.-W., & Tausch, A. (1996). Untersuchungen mit einer deutschen Version der “Positive and Negative Affect Schedule” (PANAS). [Investigations with a German version of the Positive and Negative Affect Schedule (PANAS).]. *Diagnostica*, *42*(2), 139–156.

Martin, M., & Zimprich, D. (2003). Are Changes in Cognitive Functioning in Older Adults Related to Changes in Subjective Complaints? *Experimental Aging Research*, *29*(3), 335–352. https://doi.org/10.1080/03610730303722

Oswald, W. D., & Fleischmann, U. M. (1995). *Nürnberger-Alters-Inventar (NAI).* Hogrefe.

Pennebaker, J. W. (1997). Writing About Emotional Experiences as a Therapeutic Process. *Psychological Science*, *8*(3), 162–166. https://doi.org/10.1111/j.1467-9280.1997.tb00403.x

Pennebaker, J. W., Chung, C. K., Frazee, J., Lavergne, G. M., & Beaver, D. I. (2014). When small words foretell academic success: The case of college admissions essays. *PLoS ONE*, *9*(12), e115844. https://doi.org/10.1371/journal.pone.0115844

Risch, A. K., Taeger, S., Brüdern, J., & Stangier, U. (2013). Psychological Well-Being in Remitted Patients with Recurrent Depression. *Psychotherapy and Psychosomatics*, *82*(6), 404–405. https://doi.org/10.1159/000351808

Russell, D., Peplau, L. A., & Cutrona, C. E. (1980). The revised UCLA Loneliness Scale: Concurrent and discriminant validity evidence. *Journal of Personality and Social Psychology*, *39*(3), 472–480. https://doi.org/10.1037/0022-3514.39.3.472

Ryff, C. D. (1989). Happiness is everything, or is it? Explorations on the meaning of psychological well-being. *Journal of Personality and Social Psychology*, *57*(6), 1069–1081. https://doi.org/10.1037/0022-3514.57.6.1069

Sabourin, S., Valois, P., & Lussier, Y. (2005). Development and Validation of a Brief Version of the Dyadic Adjustment Scale With a Nonparametric Item Analysis Model. *Psychological Assessment*, *17*(1), 15–27. https://doi.org/10.1037/1040-3590.17.1.15

Schulz, U., & Schwarzer, R. (2003). Soziale Unterstützung bei der Krankheitsbewältigung: Die Berliner Social Support Skalen (BSSS). [Social Support in Coping with Illness: The Berlin Social Support Scales (BSSS).]. *Diagnostica*, *49*(2), 73–82. https://doi.org/10.1026/0012-1924.49.2.73

The Whoqol Group. (1998). Development of the World Health Organization WHOQOL-BREF Quality of Life Assessment. *Psychological Medicine*, *28*(3), 551–558. https://doi.org/10.1017/S0033291798006667

Ware, J., Kosinski, M., & Keller, S. D. (1996). A 12-Item Short-Form Health Survey: Construction of scales and preliminary tests of reliability and validity. *Medical Care*, *34*(3), 220–233. https://doi.org/10.1097/00005650-199603000-00003

Watson, D., Clark, L. A., & Tellegen, A. (1988). Development and validation of brief measures of positive and negative affect: The PANAS scales. *Journal of Personality and Social Psychology*, *54*(6), 1063–1070. https://doi.org/10.1037/0022-3514.54.6.1063

**Section B – Descriptive Information**

| **Table S2**  *Descriptive Statistics: Language Variables* | | | | |
| --- | --- | --- | --- | --- |
| Variable name | Mean | Std. Deviation | Minimum | Maximum |
|  |  |  |  |  |
| Reward | 0.99 | 0.84 | 0.00 | 5.60 |
| Affect | 8.77 | 2.29 | 2.66 | 18.52 |
| I-pronouns | 6.57 | 3.85 | 0.00 | 16.11 |
| Cognitive processes | 21.01 | 3.61 | 9.25 | 32.79 |
| Quantifiers | 3.39 | 1.45 | 0.00 | 8.58 |
| Interrogatives | 1.28 | 0.81 | 0.00 | 4.05 |
| Analytical thinking | 35.79 | 28.67 | 1.00 | 99.00 |
| Affiliation | 2.25 | 1.64 | 0.00 | 15.81 |
| Family | 1.15 | 1.08 | 0.00 | 6.31 |
| Friends | 0.69 | 0.66 | 0.00 | 5.26 |
| *Note. N* = 701. All variables represent categories of the text analysis program DE-LIWC2015. Except for Analytic, all variables are percentages of the total words used in text file. Analytical thinking (see Pennebaker et al., 2014) is a composite score ranging from 0 (very low) to 100 (very high). | | | | |

| **Table S3**  *Descriptive Statistics: Questionnaire Variables* | | | | |
| --- | --- | --- | --- | --- |
| Variable name | Mean | Std. Deviation | Minimum | Maximum |
|  |  |  |  |  |
| Psychological Health (PSYCH; WHO QoLBref) | 69.93 | 18.91 | 0.00 | 100.00 |
| Life Satisfaction (current; LSSA) | 3.28 | 0.61 | 1.33 | 4.00 |
| Depression (PHQ9) | 6.39 | 4.84 | 0.00 | 24.00 |
| Life Satisfaction (SWLS) | 4.80 | 1.37 | 1.00 | 7.00 |
| Self-Acceptance (SARYFF) | 4.50 | 1.10 | 1.00 | 6.00 |
| Life Satisfaction (retrospective; LSSP) | 2.98 | 0.71 | 1.00 | 4.00 |
| Life Satisfaction (prospective; LSSZ) | 2.88 | 0.72 | 1.00 | 4.00 |
| Mental Health (MHI4P; SF-12) | 4.70 | 1.14 | 1.00 | 6.00 |
| Quality of Life (FQOL) | 24.26 | 4.62 | 11.00 | 35.00 |
| Environment (ENVIR WHO QoLBref) | 78.15 | 15.30 | 6.25 | 100.00 |
| Vitality (VITAL2; SF-12) | 3.71 | 1.16 | 1.00 | 6.00 |
| Environmental Mastery (EMRYFF; RYFF Scale) | 4.60 | 0.87 | 1.33 | 6.00 |
| Mental Health (MHI3; SF-12) | 4.23 | 1.04 | 1.00 | 6.00 |
| Positive Affect (PA; PANAS) | 3.60 | 0.74 | 1.40 | 5.00 |
| Quality of Life (SEIQoL) | 73.65 | 18.97 | 1.10 | 100.00 |
| Social Relationships (SOCIAL; WHO QoLBref) | 65.13 | 21.65 | 0.00 | 100.00 |
| Social Functioning (SOC2P; SF-12) | 4.22 | 1.02 | 1.00 | 5.00 |
| Loneliness (UCLA) | 17.50 | 8.37 | 10.00 | 48.00 |
| Positive Relationships (PRORYFF; RYFF Scale) | 4.32 | 1.06 | 1.00 | 6.00 |
| Perceived Social Support (Instrumental; BSSSI) | 3.39 | 0.66 | 1.00 | 4.00 |
| Perceived Social Support (Emotional; BSSSE) | 3.43 | 0.62 | 1.25 | 4.00 |
| Personal Growth (PGRYFF; RYFF Scale) | 5.11 | 0.75 | 2.00 | 6.00 |
| Negative Affect (NA; PANAS) | 1.83 | 0.67 | 1.00 | 4.80 |
| Global Quality of Life (OVERALL; WHO QoLBref) | 72.15 | 19.94 | 0.00 | 100.00 |
| Physical Health (PHYS; WHO QoLBref) | 78.51 | 16.20 | 10.71 | 100.00 |
| Physical Health (GHP1; SF-12) | 3.50 | 0.87 | 1.00 | 5.00 |
| Sleep Quality (JENKINS) | 4.38 | 1.11 | 1.00 | 6.00 |
| Pain (PAIN2P; SF-12) | 4.52 | 0.81 | 1.00 | 5.00 |
| Physical Functioning (PFI02P; SF-12) | 2.84 | 0.43 | 1.00 | 3.00 |
| Physical Functioning (PFI04P; SF-12) | 2.84 | 0.41 | 1.00 | 3.00 |
| *Note. N* = 701. | | | | |

| **Table S4**  *Intercorrelations Among Language Variables* | | | | | | | | | | |
| --- | --- | --- | --- | --- | --- | --- | --- | --- | --- | --- |
|  | 1 | 2 | 3 | 4 | 5 | 6 | 7 | 8 | 9 | 10 |
| 1. Reward | – |  |  |  |  |  |  |  |  |  |
| 2. Affect | .251^**^ | – |  |  |  |  |  |  |  |  |
| 3. I-Pronouns | -.135^**^ | -.069 | – |  |  |  |  |  |  |  |
| 4. Cognitive Processes | .078^*^ | .046 | .003 | – |  |  |  |  |  |  |
| 5. Quantifiers | -.062 | -.024 | .003 | .181^**^ | – |  |  |  |  |  |
| 6. Interrogatives | .008 | -.110^**^ | -.031 | .237^**^ | .020 | – |  |  |  |  |
| 7. Analytical Thinking | .078^*^ | .001 | -.365^**^ | -.402^**^ | -.276^**^ | -.234^**^ | – |  |  |  |
| 8. Affiliation | -.003 | -.004 | -.088^*^ | -.276^**^ | -.012 | -.075^*^ | .079^*^ | – |  |  |
| 9. Family | -.007 | -.025 | .129^**^ | -.248^**^ | .008 | -.210^**^ | -.030 | .512^**^ | – |  |
| 10. Friends | -.052 | .083^*^ | -.074^*^ | -.104^**^ | .001 | -.098^**^ | .131^**^ | .342^**^ | .139^**^ | – |
| * *p* < .050, ** *p* < .010, *** *p* < .001 | | | | | | | | | | |

**Section C: Detailed Overview of Results**

| **Table S5**  *Standardized Factor Loadings in Model 2 (Questionnaire Measurement Model)* | | |
| --- | --- | --- |
|  | **β [95% CI]** | |
| **Variable** | **Psychological Well-Being** | **Physical Health** |
| Psychological Health (WHO QoLBref) | 0.92*** (0.01) [0.91, 0.94] |  |
| Life Satisfaction (current) | 0.87*** (0.01) [0.84, 0.89] |  |
| Depression (PHQ9) | -0.83*** (0.01) [-0.86, -0.81] |  |
| Life Satisfaction (SWLS) | 0.82*** (0.01) [0.79, 0.85] |  |
| Self-Acceptance (Ryff scale) | 0.81*** (0.01) [0.78, 0.83] |  |
| Life Satisfaction (retrospective) | 0.79*** (0.02) [0.76, 0.82] |  |
| Life Satisfaction (prospective) | 0.79*** (0.02) [0.75, 0.82] |  |
| Mental Health (MHI4P; SF-12) | 0.76*** (0.02) [0.73, 0.80] |  |
| Quality of Life (FQOL) | 0.74*** (0.02) [0.71, 0.78] |  |
| Environment (WHO QoLBref) | 0.72*** (0.02) [0.68, 0.76] |  |
| Vitality (SF-12) | 0.70*** (0.02) [0.67, 0.74] |  |
| Environmental Mastery (Ryff Scale) | 0.69*** (0.02) [0.64, 0.73] |  |
| Mental Health (MHI3; SF-12) | 0.64*** (0.02) [0.59, 0.68] |  |
| Positive Affect (PANAS) | 0.64*** (0.02) [0.59, 0.68] |  |
| Quality of Life (SEIQoL) | 0.64*** (0.02) [0.60, 0.69] |  |
| Social Relationships (WHO QoLBref) | 0.64*** (0.02) [0.59, 0.69] |  |
| Social Functioning (SF-12) | 0.64*** (0.02) [0.59, 0.69] |  |
| Loneliness (UCLA) | -0.63*** (0.02) [-0.67, -0.58] |  |
| Positive Relationships (Ryff Scale) | 0.55*** (0.03) [0.50, 0.60] |  |
| Perceived Social Support (Instrumental; BSSSI) | 0.51*** (0.03) [0.46, 0.57] |  |
| Perceived Social Support (Emotional; BSSSE) | 0.49*** (0.03) [0.44, 0.55] |  |
| Personal Growth (Ryff Scale) | 0.38*** (0.03) [0.32, 0.45] |  |
| Negative Affect (PANAS) | -0.59*** (0.03) [-0.64, -0.54] |  |
| Global Quality of Life (WHO QoLBref) |  | 0.89*** (0.01) [0.86, 0.91] |
| Physical Health (WHO QoLBref) |  | 0.84*** (0.02) [0.81, 0.87] |
| Physical Health (GHP1; SF-12) |  | 0.77*** (0.02) [0.73, 0.80] |
| Sleep Quality (Jenkin's scale) |  | 0.53*** (0.03) [0.47, 0.59] |
| Pain (SF-12) |  | 0.42*** (0.03) [0.36, 0.49] |
| Physical Functioning (PFI02P; SF-12) |  | 0.37*** (0.04) [0.30, 0.44] |
| Physical Functioning (PFI04P; SF-12) |  | 0.34*** (0.04) [0.27, 0.41] |
| *Note.* Psychological well-being and physical health are latent variables. CI = Confidence interval. All variables had been mean-centered prior to inclusion in the model.  *** *p* < .001. | | |

**Figure S1**

*Model 2: Questionnaire Measurement Model (Schematic Illustration)*





*Note.* Residual variances were allowed to correlate for conceptually similar scales measuring the same construct (e.g., life satisfaction scales; not depicted for readability). Abbreviations of scale names are explained in Table S5.

| **Table S6**  *Standardized Factor Loadings in Model 3 (Combined Path Model)* | | | | | |
| --- | --- | --- | --- | --- | --- |
| **Variable** | ***Affective*** | ***Evaluative*** | ***Social*** | **Psychological well-being** | **Physical health** |
|  | **β [95% CI]** | | | | |
| *Reward* | 0.94** (0.36) [0.24, 1.65] |  |  |  |  |
| *Affect* | 0.27* (0.11) [0.05, 0.48] |  |  |  |  |
| *I-pronouns* | -0.10 (0.06)  [-0.21, 0.01] |  |  |  |  |
| *Cognitive processes* |  | 1.00 (.00)  [1.00, 1.00] |  |  |  |
| *Analytical thinking* |  | -0.40*** (0.03)  [-0.46, -0.34] |  |  |  |
| *Interrogatives* |  | 0.24*** (0.04) [0.17, 0.31] |  |  |  |
| *Quantifiers* |  | 0.18*** (0.04) [0.11, 0.25] |  |  |  |
| *Affiliation* |  |  | 0.78*** (0.05) [0.68, 0.88] |  |  |
| *Family* |  |  | 0.67*** (0.05) [0.58, 0.76] |  |  |
| *Friends* |  |  | 0.43*** (0.05) [0.34, 0.52] |  |  |
| Psychological Health (WHO QoLBref) |  |  |  | 0.92*** (0.01) [0.91, 0.94] |  |
| Life Satisfaction (current) |  |  |  | 0.86*** (0.01) [0.84, 0.89] |  |
| Depression (PHQ9) |  |  |  | -0.83*** (0.01)  [-0.86, -0.81] |  |
| Life Satisfaction (SWLS) |  |  |  | 0.82*** (0.01) [0.79, 0.84] |  |
| Self-Acceptance (Ryff scale) |  |  |  | 0.80*** (0.01) [0.78, 0.83] |  |
| Life Satisfaction (retrospective) |  |  |  | 0.79*** (0.02) [0.76, 0.82] |  |
| Life Satisfaction (prospective) |  |  |  | 0.78*** (0.02) [0.75, 0.82] |  |
| Mental Health (MHI4P; SF-12) |  |  |  | 0.76*** (0.02) [0.73, 0.80] |  |
| Quality of Life (FQOL) |  |  |  | 0.74*** (0.02) [0.71, 0.78] |  |
| Environment (ENVIR WHO QoLBref) |  |  |  | 0.72*** (0.02) [0.68, 0.76] |  |
| Vitality (VITAL2; SF-12) |  |  |  | 0.70*** (0.02) [0.67, 0.74] |  |
| Environmental Mastery (Ryff scale) |  |  |  | 0.69*** (0.02) [0.64, 0.73] |  |
| Mental Health (MHI3; SF-12) |  |  |  | 0.64*** (0.02) [0.59, 0.68] |  |
| Positive Affect (PANAS) |  |  |  | 0.64*** (0.02) [0.59, 0.68] |  |
| Quality of Life (SEIQoL) |  |  |  | 0.64*** (0.02) [0.60, 0.69] |  |
| Social Relationships (WHO QoLBref) |  |  |  | 0.64*** (0.02) [0.59, 0.69] |  |
| Social Functioning (SF-12) |  |  |  | 0.64*** (0.02) [0.59, 0.69] |  |
| Loneliness (UCLA) |  |  |  | -0.63*** (0.02)  [-0.67, -0.58] |  |
| Positive Relationships (Ryff scale) |  |  |  | 0.55*** (0.03) [0.50, 0.61] |  |
| Perceived Social Support (Instrumental; BSSSI) |  |  |  | 0.51*** (0.03) [0.46, 0.57] |  |
| Perceived Social Support (Emotional; BSSSE) |  |  |  | 0.49*** (0.03) [0.44, 0.55] |  |
| Personal Growth (Ryff scale) |  |  |  | 0.38*** (0.03) [0.32, 0.45] |  |
| Negative Affect (PANAS) |  |  |  | -0.59*** (0.03)  [-0.64, -0.54] |  |
| Global Quality of Life (WHO QoLBref) |  |  |  |  | 0.89*** (0.01) [0.86, 0.91] |
| Physical Health (WHO QoLBref) |  |  |  |  | 0.84*** (0.02) [0.80, 0.87] |
| Physical Health (GHP1; SF-12) |  |  |  |  | 0.77*** (0.02) [0.73, 0.80] |
| Sleep Quality (Jenkin's scale) |  |  |  |  | 0.53*** (0.03) [0.47, 0.59] |
| Pain (SF-12) |  |  |  |  | 0.42*** (0.03) [0.36, 0.49] |
| Physical Functioning (PFI02P; SF-12) |  |  |  |  | 0.37*** (0.04) [0.30, 0.44] |
| Physical Functioning (PFI04P; SF-12) |  |  |  |  | 0.34*** (0.04) [0.27, 0.41] |
| *Note.* CI = Confidence interval. Affective, evaluative, social, psychological well-being and physical health are latent variables. All language variables are presented in italics. ** p < .050, ** p < .010, **** p *< .001* | | | | | |

| **Table S7**  *Supplemental Analysis: Language Measurement Model Across Three Different Age Groups* | | | |
| --- | --- | --- | --- |
| **Variable** | **β [95% CI]** | | |
| **Younger adults (18–40 years, *n* = 415)** | | | |
|  | **Affective** | **Evaluative** | **Social** |
| Reward | 0.50** (0.15) [0.20, 0.79] |  |  |
| Affect | 0.43*** (0.12) [0.19, 0.68] |  |  |
| I-pronouns | -0.19** (0.06) [-0.31, -0.07] |  |  |
| Cognitive processes |  | 1.00 (.00) [1.00, 1.00] |  |
| Analytical thinking |  | -0.39*** (0.03) [-0.50, -0.33] |  |
| Interrogatives |  | 0.23*** (0.04) [0.16, 0.30] |  |
| Quantifiers |  | 0.20*** (0.04) [0.13, 0.28] |  |
| Affiliation |  |  | 0.80*** (0.05) [0.70, 0.91] |
| Family |  |  | 0.61*** (0.05) [0.52, 0.70] |
| Friends |  |  | 0.44*** (0.05) [0.35, 0.54] |
| **Evaluative** | 0.04 (0.09) [-0.13,0 .21] | – | – |
| **Social** | 0.05 (0.09) [-0.13, 0.22] | -0.33*** (0.05) [-0.43, -0.23] | – |
| **Middle-aged adults (40–60 years, *n* = 120)** | | | |
|  | **Affective** | **Evaluative** | **Social** |
| Reward | 0.55** (0.18) [0.20, 0.89] |  |  |
| Affect | 0.41** (0.12) [0.16, 0.65] |  |  |
| I-pronouns | -0.18** (0.06) [-0.30, -0.06] |  |  |
| Cognitive processes |  | 1.00 (0.00) [1.00, 1.00] |  |
| Analytical thinking |  | -0.36*** (0.04) [-0.43, -0.29] |  |
| Interrogatives |  | 0.22*** (0.04) [0.15, 0.29] |  |
| Quantifiers |  | 0.20*** (0.04) [0.12, 0.27] |  |
| Affiliation |  |  | 1.00*** (0.07) [0.86, 1.14] |
| Family |  |  | 0.64*** (0.05) [0.54, 0.74] |
| Friends |  |  | 0.40*** (0.05) [0.31, 0.49] |
| **Evaluative** | -0.05 (0.19) [-0.41, 0.31] | – | – |
| **Social** | 0.28 (0.18) [-0.08, 0.64] | -0.28** (0.10) [-0.48, -0.08] | – |
| **Older adults (60–87 years, *n* = 166)** | | | |
|  | **Affective** | **Evaluative** | **Social** |
| Reward | 0.64** (0.19) [0.26, 1.02] |  |  |
| Affect | 0.42** (0.13) [0.18, 0.67] |  |  |
| I-pronouns | -0.18** (0.06) [-0.30, -0.07] |  |  |
| Cognitive processes |  | 1.00 (0.00) [1.00, 1.00] |  |
| Analytical thinking |  | -0.39*** (0.03) [-0.45, -0.32] |  |
| Interrogatives |  | 0.23*** (0.04) [0.16, 0.30] |  |
| Quantifiers |  | 0.14*** (0.03) [0.09, 0.20] |  |
| Affiliation |  |  | 0.74*** (0.05) [0.63, 0.85] |
| Family |  |  | 0.66*** (0.05) [0.56, 0.77] |
| Friends |  |  | 0.32*** (0.04) [0.24, 0.40] |
| **Evaluative** | 0.10 (0.17) [-0.24, 0.43] | – | – |
| **Social** | -0.18 (0.14) [-0.45, 0.09] | -0.34*** (0.08) [-0.49, -0.18] | – |
| *Note.* Presented are standardized estimates. CI = Confidence interval. Affective, evaluative, and social are latent variables. ** p < .050, ** p < .010, **** p *< .001* | | | |
