# Supplementary material for: When I am sixty-four… evaluating language markers of well-being in healthy aging narratives
Source: PLoS One. 2024 Apr 24;19(4):e0302103. doi: 10.1371/journal.pone.0302103 (PMC11042717; doi:10.1371/journal.pone.0302103)
Supplement: S2 File — (DOCX) [file pone.0302103.s002.docx]

| **Table S8**  *Healthy aging narratives: Example excerpts (translated from German) from texts scoring high vs. low in affective, evaluative, and social language* |
| --- |
| **Affective language (high)** |
| “[…] Aging well is when you don't always look back on your younger years and long for them. Aging well is when you are satisfied with what you have done so far and can look back on your life without regret. When you feel good about where you are and the way you are. [...] Aging well is when you are happy with your own aging body and can do the things you want to do. [...] Aging well is not being bitter when seeing younger people, nor being envy for their youthfulness. No envy, because one has lived one's own youth in such a way that aging is not an obstacle. However, aging well is also aging without fear. No fear of what time will bring, no fear of one's own changes. […]” |
| “[…] Everything has to be fun though, otherwise there is no point. It is important to keep feeling fully vital, to have as many different interests as possible, to keep discovering new things. Having fun in life is most important, there simply should not be any boredom. Curiosity must be nurtured and maintained. […]” |
| **Affective language (low)** |
| “To me, aging well actually already begins as I am writing about it. For me, aging is a process of everyday life. Each day I have the opportunity to make the best out of the day, to be my best self, yet my focus is not on perfection but simply on being better than I was the day before. […]” |
| “To me, getting older means to slow down, to mature, to become more relaxed, mindful, and attentive. In my experience, this works well as long as I accompany my everyday life on the so-called meta-level: I reflect on myself, I reflect on my thinking, I reflect on what I do and what I don't do. I am better at perceiving - even if my physical senses are diminishing. […]” |
| “[...] I want to be taken seriously as an old person, too. But that means first and foremost that I need to take myself seriously as an old person, [...].  In the case of nursing care, in the nursing home: would like to be perceived as an adult, not to be belittled (with little bug or flower stickers on my name tag on the door to my room. I am not a child anymore!!). [...]  To me, dignity in old age means: not having to optimize myself in all areas, but first of all to be able to accept myself the way I am, to be able to appreciate myself, to be able to fully accept my life path and to find personal peace in doing so. [...]” |
| **Evaluative language (high)** |
| “When I think about aging, I wish that I won’t have any health problems. I do not want to have to worry about essential things in life, such as food, housing and, of course, money. I want to be free to decide what I want to do, without health, money, or other people’s opinions hindering me. I have not had any health impairments so far and would like to see this continue as I get older. [...]” |
| “Aging well, what does that actually mean? To me, it does not only mean to be healthy. It is important to consider all aspects of life. To me, this means to take something positive with me every day, no matter in which respect. Life consists of ups and downs and if one also learns something positive from the lows or can take away, this is also part of good aging for me. In addition, one should always try to fulfill ones wishes and dreams. Even if they sometimes can’t be realized. [...]  But like I said before, all dreams and wishes cannot be fulfilled. One should always have a goal in mind, even when already being a bit older. With a positive attitude to life, many things are easier to tackle than when constantly being pessimistic. I have had to learn this over time, like almost everyone, I think. In order to age well, there are many factors that need to be taken into account.” |
| **Evaluative language (low)** |
| “[...] Things one cannot change should be accepted, that is probably an important quality to cope with aging.” |
| “[...] In my opinion, good social contacts/relationships with family (partner, siblings, children, etc.) and friends are also part of good/successful aging, because they help to avoid loneliness and to remain active. Another important aspect for me is financial security, i.e. the possibility to remain financially independent in old age and to maintain a comfortable standard of living. A final aspect that would be important to me personally: having a mission, for example a mini-job, volunteering for a charitable organization, studying, or similar activities that keep myself busy and let me be around people, and that give meaning to everyday life.” |
| **Social language (high)** |
| “A basic prerequisite for aging well is to be physically and mentally healthy. It is very nice if one can experience this phase with a partner who is also healthy. It is important to have interests and hobbies that can be pursued (preferably with a partner). It is also important to maintain contacts with acquaintances and friends. [...]” |
| “By aging well, I imagine growing old surrounded by my family. I imagine myself living with my husband on a piece of land somewhere in the countryside, far away from the traffic and the hustle and bustle. We have a big house with enough beds for our children and grandchildren to visit as often as possible. [...]  Being lonely in aging is really difficult and sad, I imagine. Once I read about a project where young students lived together with older people in an apartment and, instead of paying rent, the students go shopping with their roommates, eat together and spend some time together. I think that's a really great idea.” |
| “[...] I find it important to exercise regularly. For me it was wonderful to ski in Valais with my two children and my grandchildren on my 80th birthday, such a deep feeling arose in me, I was so happy when I skied behind my children during the first descent that I had tears in my eyes, it was wonderful. [...]” |
| **Social language (low)** |
| “[…] Throughout the course of life, I want to free myself from values and ideals that are not mine, but that I have been taught by others. Healthy aging includes in this respect, a development to own autonomous thinking and own values with corresponding autonomous self-determined actions. I would like to become absolutely independent throughout the course of my life and remain so into old age, whereby physicality again comes into play. Independence is not to be confused with isolation, but meant as autonomous coexistence with the outside world.” |
| “To me, aging well is a process of constant self-realization and self-expansion. A central point is to learn and to realize, to further develop one's own empathy and love, and to be able to transfer them to as large a spectrum of existence as possible. The older I get, the more loving and empathic I want to become. [...]” |
| *Note.* The original texts were written and analyzed in German. Presented are only excerpts from participants’ writings who gave consent for anonymously sharing their narratives. |
